# Slit-Robo expression in the leech nervous system: insights into eyespot evolution

**DOI:** 10.1186/s13578-023-01019-1

**Published:** 2023-04-03

**Authors:** Hee-Jin Kwak, Brenda I. Medina-Jiménez, Soon Cheol Park, Jung-Hyeuk Kim, Geon-Hwi Jeong, Mi-Jeong Jeon, Sangil Kim, Jung-Woong Kim, David A. Weisblat, Sung-Jin Cho

**Affiliations:** 1 Department of Biological Sciences and Biotechnology, College of Natural Sciences, Chungbuk National University, Cheongju, Chungbuk 28644 Republic of Korea; 2 Department of Life Science, College of Natural Sciences, Chung-Ang University, Seoul, 06974 Republic of Korea; 3 National Institute of Biological Resources, Environmental Research Complex, Incheon, 22689 Republic of Korea; 4 Department of Molecular and Cell Biology, University of California, 385 Weill Hall, Berkeley, CA 94720-3200 USA; 5 Department of Ecology, Evolution and Behavior, Faculty of Science, Alexander Silberman Institute of Life Sciences, Hebrew University of Jerusalem, 9190401 Jerusalem, Israel; 6 Department of Earth Sciences, Paleobiology, Geocentrum, Uppsala University, Villavägen 16, 75236 Uppsala, Sweden; 7 Wildlife Disease Response Team, National Institute of Wildlife Disease Control and Prevention, Incheon, 22689 Republic of Korea; 8 Museum of Comparative Zoology and Department of Organismic and Evolutionary Biology, Harvard University, Cambridge, MA 02138 USA

**Keywords:** Slit, Robo, Gene duplication, Axon guidance, Eyespot

## Abstract

**Background:**

Slit and Robo are evolutionarily conserved ligand and receptor proteins, respectively, but the number of *slit* and *robo* gene paralogs varies across recent bilaterian genomes. Previous studies indicate that this ligand-receptor complex is involved in axon guidance. Given the lack of data regarding Slit/Robo in the Lophotrochozoa compared to Ecdysozoa and Deuterostomia, the present study aims to identify and characterize the expression of Slit/Robo orthologs in leech development.

**Results:**

We identified one *slit* (*Hau-slit*), and two *robo* genes (*Hau-robo1* and *Hau-robo2*), and characterized their expression spatiotemporally during the development of the glossiphoniid leech *Helobdella austinensis*. Throughout segmentation and organogenesis, *Hau-slit* and *Hau-robo1* are broadly expressed in complex and roughly complementary patterns in the ventral and dorsal midline, nerve ganglia, foregut, visceral mesoderm and/or endoderm of the crop, rectum and reproductive organs. Before yolk exhaustion, *Hau-robo1* is also expressed where the pigmented eye spots will later develop, and *Hau-slit* is expressed in the area between these future eye spots. In contrast, *Hau-robo2* expression is extremely limited, appearing first in the developing pigmented eye spots, and later in the three additional pairs of cryptic eye spots in head region that never develop pigment. Comparing the expression of *robo* orthologs between *H. austinensis* and another glossiphoniid leech, *Alboglossiphonia lata* allows to that *robo1* and *robo2* operate combinatorially to differentially specify pigmented and cryptic eyespots within the glossiphoniid leeches.

**Conclusions:**

Our results support a conserved role in neurogenesis, midline formation and eye spot development for Slit/Robo in the Lophotrochozoa, and provide relevant data for evo-devo studies related to nervous system evolution.

**Supplementary Information:**

The online version contains supplementary material available at 10.1186/s13578-023-01019-1.

## Background

The nervous system is a major organ system in nearly all metazoans, with the exception of sponges and placozoans [1]. Nervous system development in animals, as divergent as flies and mice, shares many features, including the evolutionarily conserved systems of ligands and receptors, such as the Slit/Robo system, that serve as guidance cues in patterning their respective nerve cords. Such commonalities have been taken as evidence that their last common ancestor (Urbilateria) presumably already had a clearly differentiated nerve cord [2]. On the other hand, a wide range of neural architectures presented by basally branching bilaterians—such as Xenacoelomorpha, Rotifera, Nemertea and Brachiopoda, together with diverse neural patterning processes they utilize—suggest that the apparent conservation of neural patterning processes and morphology between vertebrates and insects reflects an evolutionary convergence [3]. In either case, studying the genetic basis underlying the development of nervous system across diverse metazoan lineages is essential to understanding their evolutionary history.

Slit is a large, secreted glycoprotein that was first discovered in *Drosophila*, secreted by a subset of glial cells and mediated repulsive interactions along the midline of the developing central nervous system (CNS) [4]. It consists of four leucine-rich repeat (LRR) domains and multiple EGF repeats that mediate biding to the Immunoglobulin-like (IG) domains of Roundabout (Robo) [5–8]. The Slit/Robo interaction is of high apparent affinity and evolutionarily conserved [6, 7]. *slit* exists as a single copy gene in all invertebrates examined to date [9], whereas vertebrates have three paralogs—*slit1*, *slit2*, and *slit3* [10]. On the other hand, *robo* is more variable in copy number across species: *Caenorhabditis elegans* has a single *robo* (*sax*-*3*), *Drosophila* has three (*robo1*, *robo2* and *robo3*) [11], and vertebrates have four (Dutt1/Robo1, Robo2, Robo3/Rig-1, Robo4/Magic Roundabout) [7, 12].

Slit binding has been demonstrated biochemically for most Robos, except Robo4. Functionally, Slit ligands are known to be crucial for the formation of projections from the eye to brain [12–15], and for being involved in defining the position and boundaries of optic chiasm [13]. Furthermore, Slit ligands regulate axon pathfinding along the entire tract [16], repel migrating neurons—including cerebral cortical neurons, GABAergic interneurons and olfactory interneurons [17–19]—and stimulate the elongation and branching of sensory axons [20]. Robo receptors in *Drosophila* transduce a repellent signal at the midline, and the combinatorial expression of the three Robos specifies the lateral positioning of longitudinal axon tracts [11]. Additionally, Slits and Robos are dynamically expressed in complementary patterns in the migratory pathways of neural crest cells in chicks [21]. These studies seem to suggest that this ligand-receptor complex is involved in everything that concerns the relation between neurons and midline, and that this system might have acquired additional functions compared to the urbilaterian nervous system [2].

The present study aims to identify and characterize the expression of Slit/Robo orthologs in leeches as representatives of lophotrochozoans. We found one *slit* (*Hau-slit*) and two *robo* genes (*Hau-robo1* and *Hau-robo2*) in the *Helobdella* genome, and that their transcripts are expressed dynamically in late stages of development in the leech *Helobdella austinensis*, varying both temporally and spatially along the ventral nerve cord and eyespots. Our results are consistent with a conserved role for the Slit-Robo interaction in neurogenesis, midline formation, and eyespot development in the Lophotrochozoa. Furthermore, based on comparison of the expression of *slit* and *robo* orthologs in another glossiphoniid leech, *Alboglossiphonia lata*, we provide a new insight into the molecular basis of evolutionary variation of eyespot development among leeches.

## Results

### Sequence retrieval and phylogenetic analyses

**Fig. 1:**
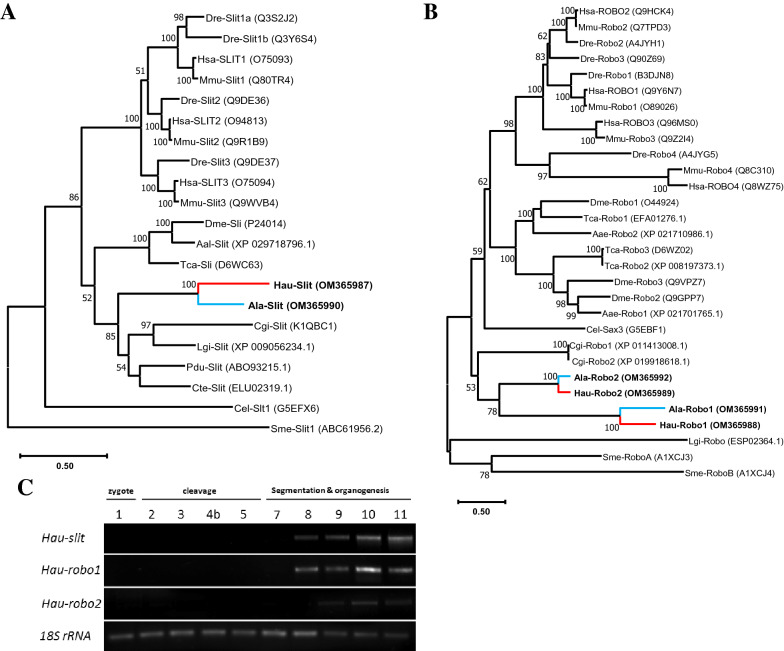
Phylogenetic analysis and temporal expression of *Slit/Robo* duplicate orthologs. **A** Phylogenetic tree for *Slit* class genes. **B** Phylogenetic tree for *Robo* receptors. **C** Semi-quantitative RT-PCR showing transcription of *Hau-slit* and *Hau-robo* orthologs. Digital images of ethidium bromide-stained agarose gels. Fragments of *Hau-slit*, *Hau-robo1*, and *Hau-robo2* were amplified in separate reactions carried out in parallel. Species abbreviations: Aae, *Aedes aegypti*; Aal, *Aedes albopictus*; Ala, *Alboglossiphonia lata*; Cel, *Caenorhabditis elegans*; Cgi, *Crassostrea gigas*; Cte, *Capitella teleta*; Dme, *Drosophila melanogaster*; Dre, *Danio rerio*; Hau, *Helobdella austinensis*; Hsa, *Homo sapiens*; Lgi, *Lottia gigantea*; Mmu, *Mus musculus*; Pdu, *Platynereis dumerilii*; Sme, *Schmidtea mediterranea*; Tca, *Tribolium castaneum*

Phylogenetic analyses for *slit* and duplicated *robo* orthologs were conducted using a dataset representing diverse metazoan species. Results from these analyses clearly indicate that the leech *slit* orthologs are members of the lophotrochozoan *slit* family supported by high bootstrap values (Fig. 1A). A molecular phylogeny of Slit protein amino acid sequences reflects the known phylogenetic relationships among vertebrates and protostomes, except *Schmidtea* (Lophotrochozoa; Platyhelminthes) and *Caenorhabditis* (Ecdysozoa; Nematoda), which appear as outliers due to long-branch attraction, as is often the case. Within protostomes, the Ecdysozoa and Lophotrochozoa are captured with modest supports, but the annelid clade (represented here by *Alboglossiphonia*, *Capitella*, *Helobdella*, and *Platynereis*) is not recovered as a monophyletic clade. A molecular phylogeny based on the Robo amino acid sequences produced similar results (except *Lottia* joining *Schmidtea* as an outlier), and indicates that the duplication of Robo occurred in the ancestor of glossiphoniid leeches after their divergence from molluscan ancestor (Fig. 1B).

### Temporal expression pattern using semi-quantitative RT-PCR

Semi-quantitative RT-PCR (sqRT-PCR) was performed to obtain a developmental expression profile for *Hau-slit*, *Hau-robo1* and *Hau-robo2* (Fig. 1C). 18S ribosomal RNA was used as an internal standard to control for variations in the efficiency of RNA extraction and reverse transcription reaction [22]. *Hau-slit and Hau-robo1* transcripts were not detected in zygotic stages prior to stage 8, indicating that these genes are zygotic, and then decreased in stage 11. *Hau-robo2* was expressed at lower levels than *Hau-slit* and *Hau-robo1*; its transcript was first detected at stage 9. Similar to *Hau-slit* and *Hau-robo1*, transcript levels for *Hau-robo2* peaked during stage 10.

### slit and robo expression patterns during development in the Leech *Helobdella*

Following the RT-PCR results described above, we used double fluorescence whole mount in situ hybridization (FWMISH) to characterize *slit* and *robo* expression patterns in *Helobdella* embryos during stage 8 to early stage 9 of development. During stage 8, the four ipsilateral ectodermal bandlets and the underlying mesoderm bandlet converge at the posterior end of the embryo to form the germinal band. The left and right germinal bands are stretched by the posterior addition of new blast cells and the oriented division of blast cell clones along the A-P axis. In late stage 8 to early stage 9, it moves circumferentially from the dorsal region and meets at the expected ventral midline of the embryo to form the germinal plate [44].

**Fig. 2:**
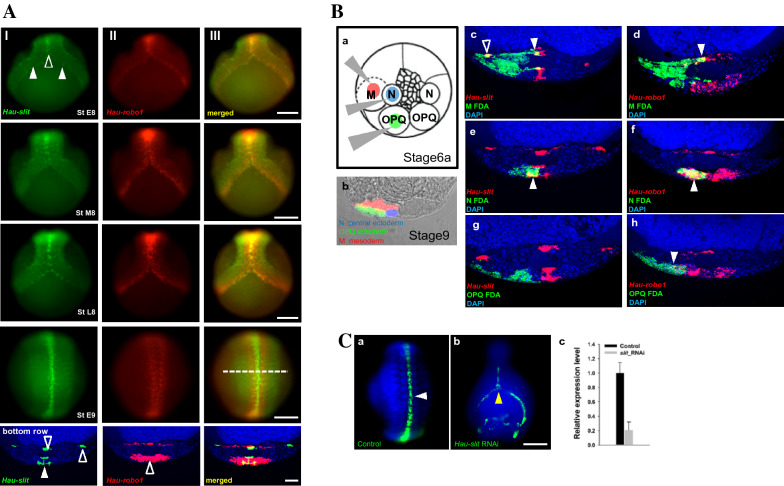
**A** Spatial expression of *Hau-slit and Hau-robo1* in *Helobdella* embryos during germinal plate formation by double FWMISH. Anterior is up in all views. Column I shows ventral view of embryos at stage 8 to early 9 expressing *Hau-slit* as longitudinally iterated dots directly apposed across the leading edges of the left and right germinal bands (white arrowhead), which coalesce zipper-like in anteroposterior progression along the future ventral midline (open arrowhead) to form a germinal plate. Column II shows ventral view of embryos at stage 8 to early 9 expressing *Hau-robo1* as longitudinally iterated dots directly apposed across the leading edges of the left and right germinal bands, similar to but broader than the pattern of *Hau-slit*. Column III shows merged image indicating the overlap of *Hau-slit* and *Hau-robo1* expression during germinal plate formation. The cross-sectional images at the bottom row show the expression region in the germinal plate at the early stage 9 embryos. Dotted lines in Column III indicate the planes of sections for the early stage 9 embryos. Yellow indicates the overlap of *Hau-slit* and *Hau-robo1* expression. *Hau-slit* expression is at the presumptive visceral mesoderm (open arrowhead), ventral and dorsal segmental ganglia (arrowhead). *Hau-robo1* expression consists of most or all cells in the segmental ganglia (open arrowhead). **B** Cell lineage analysis of *Hau-slit* and *Hau-robo1* expression. **a:** Schematic of injection strategy to identify lineage specific expression of *Hau-slit* and *Hau-robo1* transcripts (adapted from [40]). In a stage 6a embryo labeled with tracer fluorescein-conjugated dextran amine (FDA)) in the left N, OPQ, and M lineage. **b:** Pseudo-colored image (combined brightfield and fluorescence imaging) of a stage 9 embryo showing the relative positions and contributions of lineage tracer-labeled M (red), N (blue) and OPQ (green) lineages (adapted from [45]). Cross-section of stage 9 embryos showing the lineage of origin of cells expressing *Hau-slit* and *Hau-robo1* transcripts (c-h). **c:** Cross-section of an M-labeled (green) stage 9 embryo processed to reveal *Hau-slit* transcripts (red); three sets of M lineage cells expressing *Hau-slit* in lateral (open arrowhead), visceral and ventral mesoderm (white arrowhead) appear yellow. **D:** An embryo similar to that in **c**, but processed for *Hau-robo1*, reveals expression of *Hau-slit* in visceral mesoderm (white arrowhead). **E:** Cross-section of an N-labeled (green) stage 9 embryo processed to reveal *Hau-slit* transcripts (red); a set of superficial N lineage cells expressing *Hau-slit* adjacent to the ventral midline (white arrowhead) appear yellow. **F:** An embryo similar to that in **e**, but processed for *Hau-robo1*, reveals expression of *Hau-robo1* through most of the N lineage (white arrowhead). **G–h:** Cross-section of an OPQ-labeled (green) stage 9 embryos processed to reveal *Hau-slit* and *Hau-robo1* transcripts (red) shows no detectable expression of *Hau-slit* **(g)** and a few cells expressing *Hau-robo1* (**h**) in lateral ectoderm (white arrowhead). **C** Representative images of midline formation in control and *slit* knockdown embryos. **a:** *Hau-slit* transcripts are expressed in anterior to posterior germinal plate (white arrowhead) of control embryos. **b:** Knockdown of *Hau-slit* (n = 20) results in decreased expression of *Hau-slit* transcripts and disturbed coalescence of the germinal plate (yellow arrowhead). **c:** The relative mRNA expression of *Hau-slit* is shown as the mean with the standard error of the mean (SEM). The dotted line indicates the level from which cross-sections were taken. Scale bar: 100 μm in Column III; 20 μm in bottom cross-sectional image of Column III; 20 μm in Bb-g; 100 μm in Ca-b

Throughout stage 8, *Hau-slit* and its presumptive receptor *Hau-robo1* were expressed in longitudinally iterated dots, which were located at the leading edges (the prospective ventral region) of the left and right germinal bands. As the germinal bands coalesce in a zipper-like fashion in anteroposterior (A-P) progression along the future ventral midline to form a germinal plate, the dots came into direct apposition along the midline (Fig. 2A).

The cross sections of double FWMISH-stained early stage 9 embryos revealed further details of the *Hau-slit* and *Hau-robo1* expression patterns (Fig. 2A, bottom row). *Hau-slit* expression at the midline occurs primarily in three discrete levels. First, a deep layer of *Hau-slit* expressing cells appear to be in or immediately adjacent to the presumptive visceral mesoderm; lateral domains of *Hau-slit* occur at this same level, near the lateral edges of the germinal plate. Midline expression of *Hau-slit* occurs also in two other layers, which appear to mark the ventral and dorsal surfaces of the segmental ganglia, respectively. The deep, middle and superficial layers of *Hau-slit* expression are centered at the midline, but each layer is several cell diameters in width. In addition, the middle and superficial layers are connected by a narrow, dorsoventrally oriented strand of expression. *Hau-robo1* expression occurs in two broader domains that appear to complement the domains of *Hau-slit* expression. One domain of *Hau-robo1* expression consists of most or all cells in the segmental ganglia. The other domain is a thin layer of cells, apparently in visceral mesoderm, extending between the deep and lateral domains of *Hau-slit* expression.

### Germ layer localization of slit and robo expressing cells in *Helobdella* germinal plate

To examine the embryonic origin of the cells expressing *Hau-slit* and *Hau*-*robo1*, we carried out FWMISH on embryos, in which the M (mesoderm), N (primary, ventral neuro-ectoderm), and OPQ (dorsal and lateral ectoderm) lineages had been labeled by the injection of fluorescently labeled dextran amines (Fig. 2Ba, b). Both genes were expressed in subsets of cells in both the M and N lineages (Fig. 2Bc, h). *Hau-robo1* was more broadly expressed in neural lineage than did *Hau-slit* (Fig. 2Be, f)*,* and unlike the latter, it was expressed in a few cells within the OPQ lineage (Fig. 2Bg, h).

To determine whether the expression of *Hau-slit* is required for germinal plate formation, we performed a knockdown of the *Hau-slit* by injecting dsRNA into DNOPQ cells of stage 4C embryos (see Materials and Methods for details)*.* In knockdown embryos, the spatial expression of *Hau-slit* was reduced in the anterior germinal plate, and coalescence of the bilateral germinal bands into the germinal plates was disturbed compared to control embryos at late stage 9 (Fig. 2Ca, b). This observation was further supported by sqRT-PCR, which showed a decreased expression of *Hau-slit* mRNA in knockdown embryos (Fig. 2Cc). However, the disturbed coalescence of germinal bands resulted in the laceration of knockdown embryos beyond stage 9, precluding any further observation.

### slit and robo are expressed in various tissues during late development

**Fig. 3:**
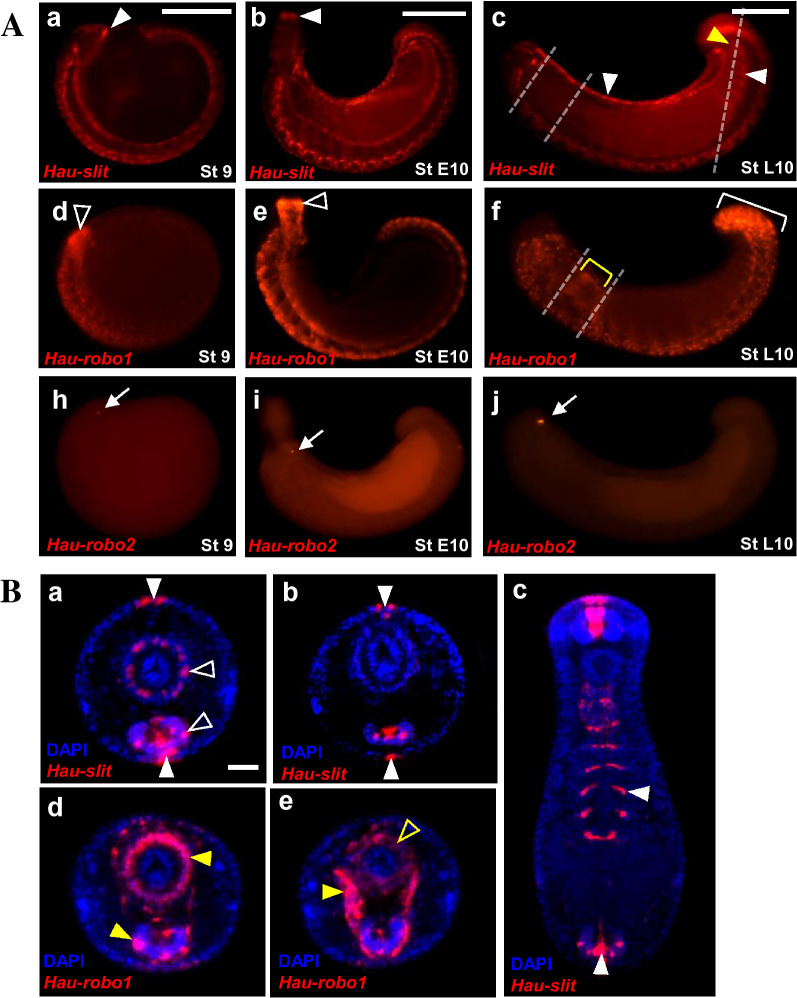
**A** Expression of *Hau-slit, Hau-robo1,* and *Hau-robo2* in *Helobdella* embryos from stages 9 to late 10 as revealed by FWMISH. Dotted lines in panels **Ac** and **Af** indicate the planes of sections for the views shown in **Ba-c** and **Bd**,**e**, respectively. Anterior is to the left in all panels. **a:** *Hau-slit* is expressed in the dorsal ganglia (white arrowhead) of the head, the developing ventral segmental ganglia and at the lateral edges of the expanding germinal plate during stage 9, with stronger expression in the anterior portion of the embryo. **b:** During stage 10, *Hau-slit* transcripts are detected along all the ventral segmental ganglia, in the anteriormost part of the everted proboscis (white arrowhead), and at the lateral edges of the germinal plate, with increasing intensity in the posterior region. **c:** At late stage 10, *Hau-slit* expression persists in the anteriormost proboscis and continues all along the ventral segmental ganglia, with greater intensity in the posterior region. As the expanding edges of the germinal plate begin to fuse dorsally, the lateral domains of *Hau-slit* expression come to occupy the dorsal midline (white arrowhead). In addition, *Hau-slit* is expressed in visceral mesoderm and/or endoderm of the intestine (white arrowhead) and muscle associated with rectum (yellow arrowhead). **d:** During stage 9, *Hau-robo1* transcripts levels are higher in the anterior to middle ventral segmental ganglia than the posterior region; expression is also seen in the developing proboscis and dorsal ganglia of the head (open arrowhead). **e:** At stage 10, *Hau-robo1* is expressed in the everted proboscis (open arrowhead) and throughout the segmental ventral ganglia, but is still stronger in the anterior half. **f:** At late stage 10, *Hau-robo1* is expressed more strongly in the posterior half of the nerve cord than in the anterior half, especially the region corresponding to the seven fused segments of the posterior sucker (bracket); also at this stage the expression has become more punctate both within the segmental ganglia and elsewhere, including the foregut (yellow bracket). **h-j:** In marked contrast to *Hau-robo1*, Hau*-robo2* is expressed selectively and with increasing abundance in the developing eyespots from stage 9 to 10. Arrow indicates eyespots. **B** Sectioned specimens reveal further details of *Hau-slit* and *Hau-robo1* expression in late stage 10 embryos. Dorsal is up in all panels. **a:** Anterior cross section shows *Hau-slit* transcripts at dorsal and ventral midline cells (white arrowhead), in subsets of ganglionic cells and in the outer ring of proboscis (open arrowhead). **b:** A more posterior cross section shows *Hau-slit* transcripts at dorsal and ventral midlines and ganglion (white arrowhead), but not in posterior proboscis. **c:** A tangentially horizontal section through the posterior part of the embryo highlights *Hau-slit* expression in ganglia, ventral midline of the body wall, and in visceral mesoderm and/or endoderm of the posterior midgut (intestine, white arrowhead). **d:** Anterior cross section reveals prominent ring of *Hau-robo1* expression in the outer portion of the anterior proboscis (yellow arrowhead), and in subsets of ganglionic cells (yellow arrowhead). **e:** Cross section through reproductive segments shows *Hau-robo1* transcripts in a U-shaped pattern corresponding to the reproductive organs (yellow arrowhead), as well as proboscis musculature and ganglia (yellow open arrowhead). Scale bar: 200 μm in A; 20 μm in B

Throughout stage 9, *Hau-slit* is expressed in dorsal ganglia of the head and developing ventral segmental ganglia (Fig. 3Aa). Meanwhile, *Hau-robo1* is expressed in dorsal ganglia of the head and in developing ventral segmental ganglia, but the transcript levels are higher in the anterior to middle ventral segmental ganglia than in the posterior region from mid to late stage 9 (Fig. 3Ad).

At stage 10, the expression of *Hau-slit* persisted along the entire ventral segmental ganglia and the anteriormost part of the developing everted proboscis, as well as in the dorsal midline (Fig. 3Ab). At late stage 10, *Hau-slit* is expressed along the entire ventral segmental ganglia, dorsal midline and rectum (Fig. 3Ac). At early-to-mid stage 10, *Hau-robo1* is expressed strongly along all the proboscis and segmental ventral ganglia (Fig. 3Ae). At late stage 10, *Hau-robo1* is expressed in a pattern of sequential dots along the entire ventral segmental ganglia, and as dots scattered all over the foregut, which surrounds the invaginated proboscis and midgut. Furthermore, *Hau*-*robo1* is expressed strongly in the caudal region (Fig. 3Af).

Consistent with the low overall levels of *Hau-robo2* detected in our RT-PCR analysis, *Hau-robo2* is expressed in a highly restricted pattern, which corresponds to a bilateral pair of anterodorsal spot gradually increasing in intensity from stage 9 to 10 (Fig. 3Ah-j).

For a more detailed view of the *Hau-slit* and *Hau-robo1* expression, we examined stained late stage 10 embryos in section (Fig. 3B). The examination of serially sectioned material revealed that *Hau-slit* is expressed in a sparsely distributed set of cells in the radial muscle of the proboscis. In addition, *Hau-slit* transcripts are detected in visceral mesoderm and/or endoderm of the intestine (Fig. 3Ba-c). On the other hand, *Hau-robo1* is expressed in the outer ring of the proboscis sheath—i.e., longitudinal muscle of the proboscis, radial muscle precursor, and ventral segmental ganglia, as well as in a precursor of presumptive reproductive organs [22] (Fig. 3Bd-e).

The pattern of *Hau-slit* expression observed within segmental tissues at the end of stage 8 has been transformed by late stage 10 as follows: Within the deep layer, *Hau-slit* expression in the midline appears to have disappeared (compare Fig. 2AI bottom row to Fig. 3Bb). Furthermore, the transcript domains near the lateral edges of the germinal plate meet at the dorsal midline, reflecting the spreading of the germinal plate around the circumference of the yolk to generate the body tube from stage 9 to 10 [44]. The superficial layer of expression is reduced to a narrow domain of midline cells in the body wall. Moreover, the middle layer of *Hau-slit* expression is now comprised of a few bilateral pairs of cells within the ganglia and at its dorsal surface (Fig. 3Bc).

### Ganglionic expression of slit and robo

**Fig. 4:**
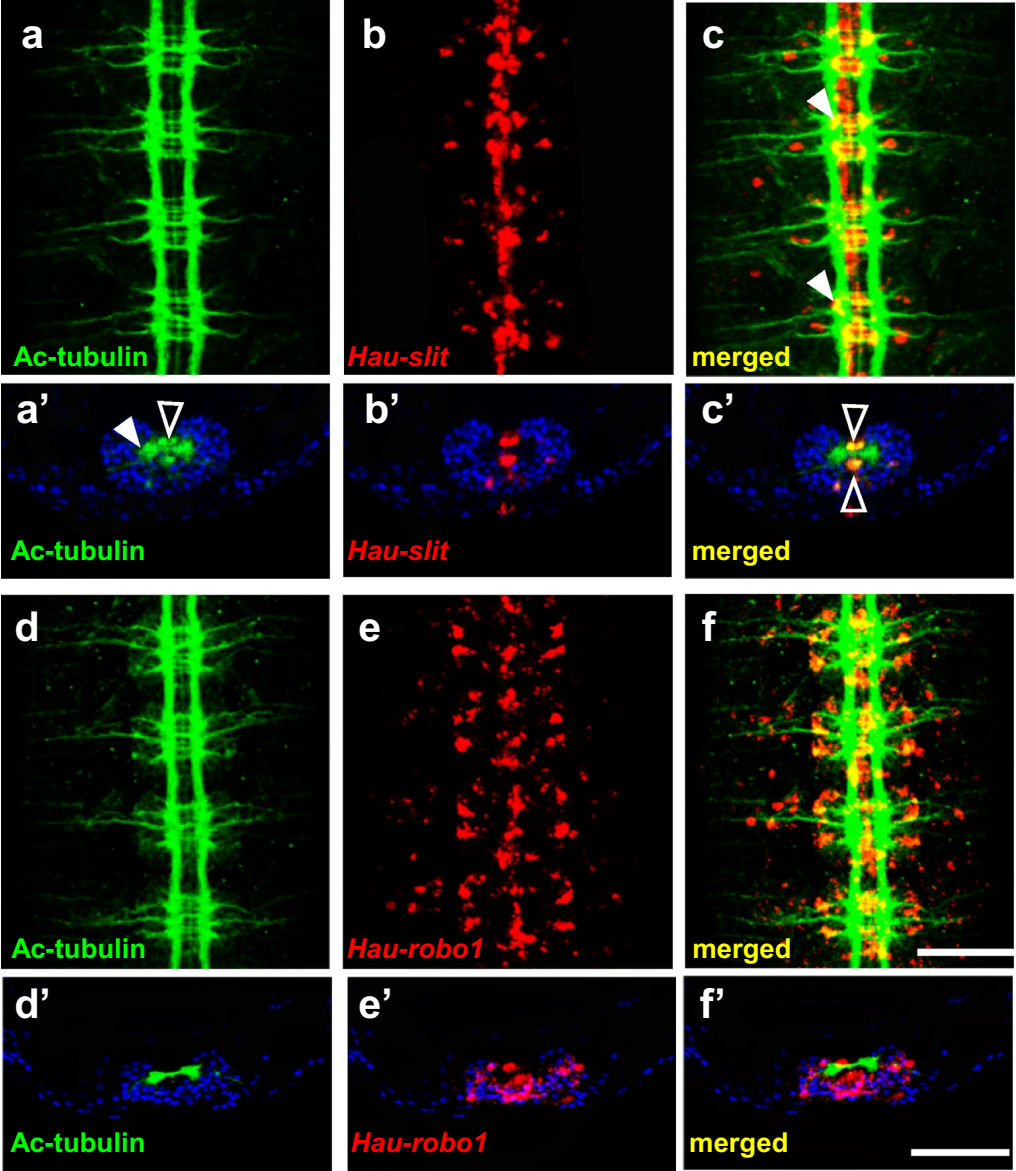
Immunostaining for acetyl-tubulin (ac-tubulin) provides landmarks for the regions within the segmental ganglia where *Hau-slit* and *Hau-robo1* are expressed. **a:** Ventral view showing ac-tubulin staining of the CNS. **a':** Cross section of stage 10 embryo showing ac-tubulin staining in neuropil (open arrowhead) and commissures (white arrowhead). **b:** Ventral view showing *Hau-slit* expression. **b':** Cross section of stage 10 embryo showing *Hau-slit* staining neuropil partially. **c:** Merged image showing *Hau-slit* transcripts overlapping partially with commissures (white arrowhead). **c':** Merged image of both cross sections showing *Hau-slit* transcripts at the ventral and dorsal margins of the neuropil (open arrowhead). **d:** Ventral view showing ac-tubulin staining of the CNS. **d':** Cross section of stage 10 embryo showing ac-tubulin staining in neuropil and commissures. **e:** Ventral view showing *Hau-robo1* expression. **e’:** Cross section of stage 10 embryo showing *Hau-robo1* staining. **f:** Merged image showing *Hau-robo1* transcripts surrounding every ganglia and overlapping partially with the segmental nerves, commissures, neuropil and the connective. **f':** Merged image of both cross sections showing *Hau-robo1* transcripts along the midline in between each hemiganglia and overlapping partially with commissures, neuropil and the connective. Anterior is up in a-c and d-f; dorsal is up in a'-c' and d'-f'. Scale bar: 100 μm in a-f; 50 μm in a'-f'

To examine the ganglionic expression of *Hau-slit* and *Hau-robo1* in detail, we combined FWMISH for *Hau-slit* and *Hau-robo1* with immunostaining of acetylated-α-tubulin to mark developing axon tracts in stage 10 embryos. As previously described in another glossiphoniid leech species [23], immunostaining revealed prominent bilaterally paired longitudinal axon tracts of connective nerves, three main pairs of segmental nerves (previously named AA, MA and PP) exiting each ganglion, and three main commissures in the neuropil of each ganglion (Fig. 4a, d). The longitudinal connectives and neuropil are dorsally situated, with cell bodies on the ventral and lateral aspects of the ganglia (Fig. 4a', d'). The patterns revealed by a combined immunostaining of acetylated-α-tubulin and FWISH for *Hau-slit* and *Hau-robo1* are consistent with the conclusion that these genes are expressed in specific subsets of ganglionic cells. For *Hau-slit*, we observe a pair of prominent cells flanking the ventral midline between the MP and PP nerves (Fig. 4b, c). At the midline itself, *Hau-slit* is expressed in two bilateral pairs of cells on the dorsal side of the neuropil and one pair of anteroposteriorly arrayed cells on the ventral side of the neuropil (Fig. 4a’-c’ and data not shown). Based on our lineage tracing analysis (Fig. 2Bb and Bd), we found the previously described M lineage-derived connective muscle cells and N lineage-derived neuropil glia as likely candidates for the source of this expression [24]. *Hau-robo1* is expressed in pairs of relatively large cells in the interstices separating the three segmental nerves on each side of the ganglia, and smaller cells positioned along the segmental nerves in the periphery (Fig. 4e, f).

### slit and robo expressed in the eyespots

**Fig. 5:**
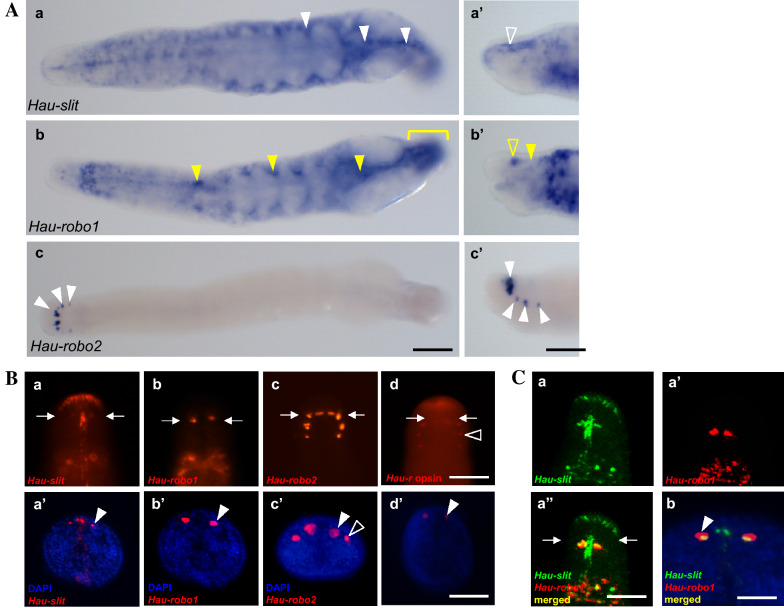
**A** Expression of *Hau-slit, Hau-robo1,* and *Hau-robo2* in *Helobdella* embryos at late stage 11. FWMISH was carried out before the appearance of pigmentation in the eyespots. All embryos show dorsal view and are oriented anterior to left. **Aa’, Ab’** and **Ac’** indicate the lateral view of the head region shown in **Ba**-**c**. **a:** *Hau-slit* is expressed in the middle line of the head and the developing ventral segmental ganglia with greater intensity in the body region. In addition, *Hau-slit* expression is seen in visceral mesoderm and/or endoderm of the intestine and muscle associated with rectum (white arrowhead). **a’**: In the magnified lateral view of head region, the expression of *Hau-slit* is clearly visible as a linear patch in cerebral ganglia (open arrowhead). **b:** *Hau-robo1* is expressed in the nerve cord, visceral mesoderm and/or mesoderm of the intestine, presumptive primordial germline (yellow arrowhead), and the region corresponding to the seven fused segments of the posterior sucker (yellow bracket). **b’:** In the magnified lateral view of head region, *Hau-robo1* is expressed in the eye precursor (yellow open arrowhead) and cerebral ganglia (yellow arrowhead). **c–c’:** *Hau-robo2* is expressed selectively in the developing eyespots (white arrowhead). **B** Differential expression pattern between *Hau-slit* and duplicated *Hau-robo* orthologs in *Helobdella* embryos at late stage 11 using fluorescent in situ hybridization. DAPI staining was performed to visualize cross sectional morphology by nuclei labeling. White arrows indicate sectional region. **a:** Dorsal view of head region of late stage 11 embryo showing *Hau-slit* staining the eyespots (white arrowhead) and in a narrow longitudinal patch of tissue between them. **b:** Dorsal view of late stage 11 embryo showing *Hau-robo1* staining the eyespot (white arrowhead). **c:** *Hau-robo2* transcripts are detected in the eyespots (white arrowhead) and phaosomes (open arrowhead). **d:** *Hau-r opsin* was used as a marker to compare the differential expression patterns in eyespots and phaosomes (open arrowhead). Dorsal view of head region of late stage 11 embryo showing *Hau-r opsin* staining the eyespots (white arrowhead). **C** Co-localization of *Hau-slit* and *Hau-robo1* transcripts. **a”-b:** Overlapped expressions of *Hau-slit* and *Hau-robo1* transcripts are shown in eyespots (white arrowhead). Scale bar: 75 ​μm in Aa-c; 50 μm in Aa'-c'; 50 μm in Ba-d; 30 µm in Ba'-d'; 50 μm in Ca-a''; 20 µm in Cb

By late stage 11, *Hau-slit* is expressed at the margin of the anterior sucker, in eyespots, and as a narrow longitudinal patch between the eyespots, which corresponds to cerebral ganglia (Fig. 5Aa-a’ and 5Ba). It is also expressed in the developing ventral segmental ganglia, in visceral mesoderm and/or endoderm of the muscle associated with the rectum, and strongly in the body region (Fig. 5Aa). *Hau-robo1* is expressed in a single bilaterally paired set of cells in the anterior (head) region, corresponding to the pigmented eyespots (Fig. 5Ab, b', and Bb, b'). It is also expressed in the nerve cord, visceral mesoderm, presumptive primordial germline, and in the posterior sucker (Fig. 5Ab). Consistent with the results obtained with single probes, the expression of *Hau-slit* and *Hau-robo1* partially overlaps in the eyespots in these late stage 11 specimens (Fig. 5Ca”, b).

In contrast, *Hau-robo2* is expressed in four bilaterally paired sets of cells in the region that corresponds to a developing pair of pigmented eyespots and three pairs of extra ocular photoreceptor cells (PRCs) (Fig. 5Ac, c’ and Bc). All four pairs of eyespots express *r-opsin* transcripts during late stage 11 (Fig. 5Bd, d'), as reported by [25].

### Eyespots by robo2 expression in leeches

**Fig. 6:**
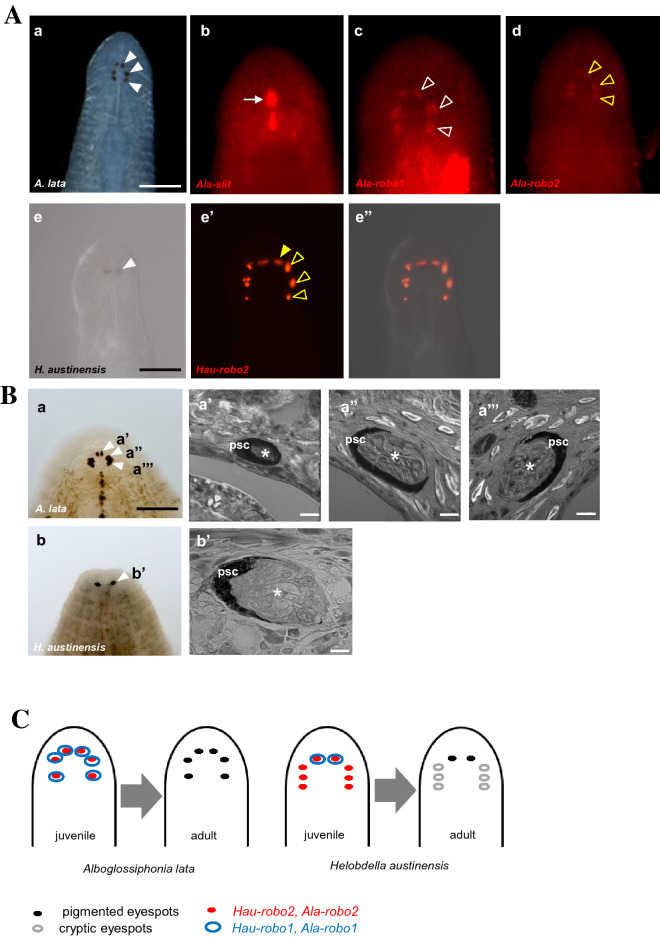
**A** Expression patterns of Slit/Robo orthologs in *Alboglossiphonia lata* embryos using fluorescent in situ hybridization. FWMISH was carried out after the appearance of pigmentation in the eyespots. Anterior is up in all views unless indicated otherwise. Asterisks indicate photoreceptor cells (PRCs). **a:** Head region of *A. lata* juvenile showing the 3 pairs of eyespots (white arrowhead). **b:** *Ala-slit* expression pattern in a pair of longitudinal narrow patches (white arrow) between eyespots. **c:** *Ala-robo1* is expressed in presumptive photoreceptor cells of 3 pairs of eyespots (open arrowhead). **d:** *Ala-robo2* is expressed in presumptive pigmented supporting cells of 3 pairs of eyespots (open yellow arrowhead). **e**: Head region of *Helobdella austinensis* juvenile showing the 1 pair of eyespots (white arrowhead). **e’**-**e’’**: *Hau-robo2* was used as a marker to compare the expression pattern of transcripts of the *Alboglossiphonia lata robo2* ortholog. *Hau-robo2* is expressed in the region of photoreceptor cells (PRCs, yellow arrowhead) including extra ocular PRCs (open yellow arrowhead). Scale bar: 100 μm in a-d; 100 μm in e-e'' **B** Pseudocolorized laser scanning microscopic (LSM) view of histological sections of adult *A. lata* and *H. austinensis*. **a**: *A. lata* has 3 pairs of eyespots with the first pair located closely each other. **a’-a’’’**: Internal structure of *A. lata* eyespots is composed of pigmented supportive cells (PSCs) and phaosomal photoreceptor cells (PRCs, asterisk). **b**: 1 pair of eyespots is clearly visible in anterior head of *H. austinensis*. **b’**: pigmented eyespot of *H. austinensis* is composed of PSCs and phaosomal photoreceptor cells (PRCs, asterisk). Scale bar: 100 μm in a-b; 10 μm in a-a''', b'. **C** Schematic summary of the combinatorial expression patterns by which *robo* genes are proposed to specify pigmented and cryptic eyespots in two glossiphoniid leech species

Unlike *Helobdella* showing a single pair of pigmented eyespots (Fig. 6Ae), another glossiphoniid leech species, *Alboglossiphonia lata* (*Ala*), is characterized by having three pairs of pigmented eyespots (Fig. 6Aa). This interspecific difference makes the comparison of *slit* and *robo* orthologs in the two species particularly interesting. In fact, we found some notable similarities and differences in the expression of *Ala-slit* relative to its ortholog in *Helobdella*. First, we observed *slit* expression in a pair of longitudinally orientated midline patches of tissue between the multiple eyespots in *Alboglossiphonia* (Fig. 6Ab), compared to just one in *Helobdella* (Fig. 5Ba). Second, we were unable to detect any *Ala-slit* expression within the eyespots themselves in *Alboglossiphonia*, although this failure could simply result from higher levels of autofluorescence in *Alboglossiphonia* embryos. Unfortunately, the increased autofluorescence also made it impossible to carry out double-FWMISH in this species. Intriguingly, both *Ala-robo1* and *Ala-robo2* are expressed in all three pairs of pigmented eyespots, consistent with the co-expression of both *robo* paralogs in only the pigmented eyespots in *Helobdella* (Fig. 5Bb, c)*.* In addition, we also observed the internal structure of pigmented eyespots whether they are composed of photoreceptor cells and pigmented supportive cells (PSCs) as previously described [25]. In the given section view of both species, it was corroborated that the PRCs and pigmented supportive cells were clearly visible is 3 pairs of eyespots in *A. lata* (Fig. 6Ba’-a’’’), which were structurally identical with *H. austinensis* (Fig. 6Bb, b’).

## Discussion

Here, we have identified and characterized the expression of homologs of *slit* and *robo* genes in two glossiphonid leech species, *Helobdella austinensis* and *Alboglossiphonia lata*. We found that these species contain a single *slit* gene, as do all other protostomes that have been examined (Fig. 1A) [7, 26]. In the case of *robo*, however, we found two copies in each species, which is also consistent with other protostomes where two or more *robo* paralogs are found. Intriguingly, our phylogenetic analysis indicates that *robo* duplications have occurred independently at least three times within the mollusk, flatworm and leech lineages (Fig. 1B) [27]. Together, these findings suggest that the Slit ligand is subject to more severe evolutionary constraints than the Robo receptor.

Our semi-quantitative RT-PCR results indicate the expression of *Hau-slit* and *Haurobo1* throughout the segmentation and organogenesis in *Helobdella austinensis* (Fig. 1C). This result was corroborated by FWMISH. *Hau-slit* is expressed in the leading (prospective ventral) edge of the coalescing germinal bands, and in the lateral (prospective dorsal) edge of the expanding germinal plate, suggesting its potential role in the formation of both ventral and dorsal midlines (Fig. 2AI). Both mesodermal and ectodermal lineages express *Hau-slit* and *Hau-robo1* (Fig. 2Bc, d–g, h), and knockdown of *Hau-slit* in the DNOPQ lineage demonstrates its tight association with the coalescence of germinal band during stage 8 (Fig. 2Ca, b). This result suggests a potential role of *Hau-slit* in the epibolic migration of germinal bands, similar to the previously reported role of *Hau-netrin-1* [28].

From their multiple sites of expression, *Hau-slit* and *Hau-robo1* appear to be involved in patterning both non-segmental (proboscis and gut) and segmental tissues (Fig. 3B). A rearward progressing wave of *slit* and *robo* gene expression, especially clear for *Hau-robo1*, correlates with a segmental differentiation (Fig. 3Aa–f). In contrast, the expression of *Hau-robo2* is confined to developing eyespots and associated phaosomes, indicating a derived and specialized function of this gene (Fig. 3Ah–j and Fig. 5Ac, Bc and 6Ae’).

*Hau-slit* expression in particular occurs in a narrow longitudinal pattern along both the ventral and dorsal midlines (Fig. 3Ba, b), indicating its conserved function in midline signaling, and is consistent with the role this gene plays in repelling migrating neurons that form ganglia and connectives from the midline (Fig. 4a–c). The presence of *Hau-slit* transcripts between the developing eyespots might also indicate the repelling role of this gene during eyespot formation, in which it is involved in defining the position and boundaries of these organs (Fig. 5Ba). Slit ligands are known to be involved in the formation of projections from the eye to brain, and in axon pathfinding regulation along the tract in vertebrates [13–16, 19]. It is interesting to note that in *Drosophila*, Robo receptors are associated with repelling signals at the midline [12]. Thus, studies of vertebrates and insects imply a conserved involvement of the Slit/Robo system in everything that concerns the relation between neurons and midline [2]. Not surprisingly, our observations on the expression of *slit* and *robo* genes along the developing segmental ganglia also suggest a similar role of Slit/Robo in neural patterning in the Lophotrochozoa. We speculate that the expression of *slit* and *robo* during later organogenesis, in association with proboscis, eyespots, crop and reproductive organs, may indicate that the Slit/Robo system is involved in the formation of peripheral nerves that interconnect major internal organs.

In the past, light sensitive photoreceptor cells were considered to exist in just two types—ciliary and rhabdomeric photoreceptors [29–31]. In the Annelida, however, a third cell type, the phaosomal photoreceptor, has been described, which is characterized by a dramatically infolded apical surface so that the sensory microvilli appear to occupy an internal cavity of the cell [25, 31–33]. Phaosomal photoreceptor cells are rare in the Polychaeta, but may be the only photoreceptor cell type present in the Clitellata. It has been suggested that phaosomal receptors are originally derived from rhabdomeric receptors—for example, they express rhabdomeric opsins—and that clitellate eyes have re-evolved after being lost during the evolution of clitellates from their polychaete ancestors. The loss of ancestral annelid eyes in that transition could have occurred in association with the emergence to direct development and/or the occupation of endobenthic habitats in the clitellate stem lineage [25, 31].

In the work reported here, we observed that the combinatorial gene expression of the *robo1* and *robo2* genes correlates with the distinction between pigmented and unpigmented (cryptic) eyespots of *Helobdella austinensis*. At late stage 10, *Hau-robo2* is consistently expressed in the developing pigmented eyespots (Fig. 3Aj), and subsequently in the three pairs of unpigmented, thus cryptic, eyespots at late stage 11 (Fig. 5Ab–c’, Bc). Cryptic eyespots have been previously observed in *Helobdella robusta* based on the expression patterns of *opsin* and *gq-protein* genes [25]. These results suggest that the expression of *robo2* might be critical for the development of eyespots, including the phaosomal photoreceptors. In contrast to *Hau-robo2*, *Hau-robo1* is expressed only in the pigmented eyespots of *Helobodella*, suggesting that *robo1* act as a decision maker for the development of pigmented epithelium and/or other features that are unique to pigmented eyespots (Fig. 5Ab, Bb).

The idea that *robo1* and *robo2* operate concertedly in the specification of pigmented (requiring both *robo1* and *robo2*) and unpigmented (*robo2* only) eyespots is further supported by their expression patterns in *Alboglossiphonia lata*, the second glossiphoniid leech species we examined. In contrast to *Helobdella*, which exhibits one pair of pigmented and three pairs of unpigmented eyespots, *Alboglossiphonia* presents three pairs of eyespots that are all pigmented with combination of PRCs and PSCs, in which the expression of both *robo1* and *robo2* is confirmed (Fig. 6A, B). Thus, we speculate that *robo2* is a key factor in specifying the baseline state of cryptic eyespot differentiation, and that the addition of *robo1* expression is required to specify fully differentiated pigmented eyespots (Fig. 6C).

Leeches as a group vary widely in the number and distribution of their eyespots [34]. Such a diversity in eyespot among leeches is not surprising given their long evolutionary history over ~ 360 million years [35], as well as pervasive ecological shifts in habitats and lifestyle [36]. In fact, the variation in pigmented and cryptic eyespots observed in the present study is particularly fascinating since the two species under investigation both belong to the family Glossiphoniidae, representing only one of the earliest branching leech lineages [37]. Our finding elucidates the undoubted importance of *robo* expression in the differentiation of eyespots in leeches, and set the framework for studying the genetic and developmental basis underlying the eyespot evolution across all leeches, and even annelids as a whole.

## Conclusions

Our results of the *slit* and *robo* gene characterization in *Helobdella* support their conserved role in the development of nervous system, including the midline formation and eyespot development in the Lophotrochozoa. A comparison with another glossiphonid leech, *Alboglossiphonia*, allows us to highlight the importance of differential *robo* expression in the loss of pigmented eyespot pairs across the family. Based on the results presented here, we intend to expand our investigation to other early-diverging and paraphyletic species of leeches showing intermediate characteristics to shed light on the eyespot evolution. Finally, our results provide relevant information for evo-devo studies related to nervous system evolution across Bilateria.

## Methods

### Embryos

Embryos were obtained from a laboratory colony of the species *Helobdella austinensis* and *Alboglossiphonia lata* as described elsewhere [38–41]. The specimens were cared for once daily by changing solution and the bowl was scrubbed manually to get rid of any residual waste. They were stored in a BOD incubator at 22 ℃.

### Alignment and phylogenetic analyses

Two separate Slit and Robo data set were built with respective Slit and Robo homologs from representatives of the major metazoan clades with a total of 21 amino acid sequences for Slit and 30 for Robo. Slit and Robo protein sequences were retrieved from the UniProt database and GenBank. Alignments were carried out using MUSCLE (Multiple Sequence Comparison by Log- Expectation) in implemented in MEGA X software. The aligned sequences were analyzed with MEGA X software [42] using the model selected by finding best fit models [43]. The Maximum Likelihood analyses were conducted by generating 1,000 bootstrap replicates.

### Developmental semi-quantitative RT-PCR

Total RNA samples were prepared from *H. austinensis* embryos at selected stages [44] with RNA Wiz (Ambion, Austin, TX, USA) according to the manufacturer's instructions, using 40 embryos for each stage. The total RNA obtained (3 µg) was reverse transcribed using a first-strand cDNA synthesis kit (BD Biosciences, Palo Alto, CA, USA). The PCR mixture (50 μl) contained 10 × Taq buffer, 0.3 U Taq polymerase (PerkinElmer, Wellesley, MA, USA), 2.5 mM of dNTPs, 5 pmol of each set of primers (for the details of primer information, see Additional file 1: Table S1), and 50 ng of cDNA from the selected stages as template. The PCR reactions were performed under the following cycling conditions: an initial denaturation at 94 °C for 5 min, followed by 25–35 cycles of denaturation at 94 °C for 30 s, and elongation at 72 °C for 10 min. A 6 μl aliquot of each PCR reaction was removed after 25 cycles, while the remaining material underwent five additional cycles of amplification. The 18 rRNA sequence was used as an internal standard (QuantumRNA, Ambion). The extent of amplification was chosen empirically to avoid saturation of the amplified bands. To quantify PCR products, each sample was run in a 1.5% agarose gel, and then stained with ethidium bromide. Band intensity was measured with an Alphalmager (Alpha Innotech Corp., San Leandro, CA, USA) using the Alphaease (v3.3b) program.

### Probe synthesis for in situ hybridization

To isolate the *H. austinesis slit* and *robo* gene, previously published sequences, we used BLAST implemented in the whole draft-genome reference (http://genome.jgi.doe.gov/Helro1/Helro1.home.html). Three candidate genes (protein id: *Hau-slit* 103882; *Hau-robo1* 189275; *Hau-robo2* 188972) were screened. In the *A. lata* transcriptome data, one *slit* and two *robo* transcripts were found. The *slit* and *robo* specific primers were designed to amplify the fragments (For detailed primer sequences, see Additional file 1: Table S1). These amplified fragments were cloned into pGEM T vector (Promega, Madison, WI, USA). RNAprobes labeled with digoxigenin or fluorescein were made using the MEGAscript kit (Ambion, Austin, TX, USA) and DIG or Fluorescein RNA Labeling Mix (Roche, Basel, Switzerland), according to the manufacturer's instructions. The synthesized RNA probes were applied to each sample at a final concentration of 2 ng/μl.

### Fluorescence in situ hybridization

We carried out double fluorescence whole mount in situ hybridization (FWMISH) using the NEN tyramide signal amplification (TSA) Plus kit (PerkinElmer, Wellesley, MA, USA). The protocol was identical to that in Cho et al. 2010, where stage 8 to 11 embryos were rinsed once in 1 × PBTw and blocked for 2 h at room temperature in a solution of 0.1 M Tris–HCl pH 7.5, 0.15 M NaCl, and 0.1% Tween 20 (TNT) containing 1% NEN TSA blocking reagent (collectively designated TNB). Embryos were incubated overnight at 4 °C with peroxidase-conjugated anti-digoxygenin or anti-fluorescein (Roche, Basel, Switzerland) at a 1:1000 dilution in TNB. Subsequent washes (3 × 20 min) in TNT at room temperature were followed by a single 1 × 30 min rinse in NEN TSA Plus amplification solution. The color reaction was initiated by adding a 1:50 dilution of reconstituted cyanine-3 tyramide in NEN amplification solution. For fluorescence microscopy, embryos were counterstained with 4,6-diamidino-2-phenylindole (DAPI, Sigma) to visualize cell nuclei. FWMISH was visualized using a Leica compound microscope and a spinning disk confocal microscope (Leica, Wetzlar, HE, Germany).

### Lineage tracing and cross sectioning

To determine the embryonic origin of the cells expressing specific *Hau-slit* and *Hau-robo1*, teloblasts or proteloblasts of embryos in stages 5 and 6 were injected with a fluorescently labeled, fixable dextran lineage tracer, fluorescein-conjugated dextran amine (FDA), as previously described [41]. Injected embryos were cultured in *Helobdella triserialis* saline (HTR) at 23 °C to the desired embryonic stage, then fixed, and processed by FWMISH as described above. After FWMISH, the embryos were dehydrated in ethanol and propylene oxide, followed by infiltration with plastic embedding medium (PolyBed, Polysciences, Inc.). Then embryos were sectioned by using a glass knife on a microtome (MT-2B; Sorvall, Newtown, CT, USA) or hand cut by razor blade into 0.1 mm sections. Sections were imaged on a Leica compound microscope to obtain combined fluorescence images (Leica, Wetzlar, HE, Germany).

### Hau-slit Double-Stranded RNA (dsRNA) Preparation

Single-stranded RNAs were produced from opposing strands of a full-length cDNA clone in pBluescript II (Stratagene), by in vitro transcription with the T3 and T7 polymerases (Ambion) (For the details, see Additional file 1: Table S1). The plasmid DNA was then removed by using DNaseI from the RNase-free kit (Ambion). The two single-stranded RNAs were allowed to anneal by mixing equal amounts of each strand, heating to 85 °C, and cooling gradually to 40 °C. The quality of the annealed dsRNA was checked by electrophoresis on an agarose gel. NOPQ proteloblasts injected with *Hau-slit* dsRNA at 100 ng/μl. Injected embryos were cultured in a modified HL medium (9.6 mM NaCl, 1.2 mM KCl, 2 mM MgCl2, 8 mM CaCl2, 1 mM maleic acid, pH6.6) supplied with antibiotics (0.05 mg/ml tetracycline, 100 units/ml penicillin, 100 units/ml Streptomycin).

### Immunostaining

After rehydrating stage 10 embryos, they were pre-incubated in 5% mercaptoethanol and 1% Triton in 0.1 M Tris–HCl (pH 7.5) at 37 °C on shaking incubator (rpm 60) for an hour. Following three washes with PBT, the embryos were incubated in Block solution (1:9 10X Roche Western Blocking Reagent in PBT) for two hours. Then, embryos were incubated with a monoclonal anti-acetylated-α-Tubulin antibody (Sigma, T-7451) in Blocking Solution (1:500) at 4 °C for 72 h. After three consecutive washes with PBT, embryos were incubated with a secondary antibody (Abcam, ab150113) in Blocking Solution (1:1000) at 4 °C for 48 h. Consequently, embryos were washed with PBT overnight. Then embryos were stained with DAPI in PBT (1:1000) at room temperature in the dark overnight. After washing with PBT 3 more times, embryos were embedded in 30%, 50% 20 min and 87% glycerol and 2.5 mg/ml of DABCO in 1xPBS. Embryos were imaged by fluorescence microscopy on a Nikon SMZ18 Stereomicroscope and LEICA DM6 B.

## Supplementary Information

**Additional file 1: Table S1.** Specific primers used for in situ hybridization, sqRT-PCR, and RNAi.

## Data Availability

Related sequencing data have been uploaded to NCBI’s GeneBank. Other data supporting the findings of the present study are available from the corresponding author upon reasonable request.
