# Supplementary material for: Slit-Robo expression in the leech nervous system: insights into eyespot evolution
Source: Cell Biosci. 2023 Apr 3;13:70. doi: 10.1186/s13578-023-01019-1 (PMC10071614; doi:10.1186/s13578-023-01019-1)
Supplement: Supplementary file 1 — Additional file 1: Table S1. Specific primers used for in situ hybridization, sqRT-PCR, and RNAi. [file 13578_2023_1019_MOESM1_ESM.docx]

**Table S1. Specific primers used for *in situ* hybridization, sqRT-PCR, and RNAi.**

| **Gene Name** | **Primer Name** | **Primer sequence** | **PCR product (bp)** | **Accession**  **number** |
| --- | --- | --- | --- | --- |
| Hau-slit  riboprobe | slit F | 5’- TTGCACAGCCTCAAAATTATAAGACTTGGT-3’ | 618 | OM365987 |
|  | slit R | 5’- AAGCAGATTGAGATTGGCGAGCCTCGAAAA-3’ |  |  |
| Hau-robo1  riboprobe | robo1 F | 5’- AACCAAACCCTACCCCAGGGTGAAACAGTC-3’ | 813 | OM365988 |
|  | robo1 R | 5’- GTATTCATCAAAGGAGCCCCATTCAACTTG-3’ |  |  |
| Hau-robo2  riboprobe | robo2 F | 5’- CACGGCCCTCAGAACCAAACCCTACCCCAG-3’ | 810 | OM365989 |
|  | robo2 R | 5’- GCCCCATTCAACTTGAATGCTTGTCGAGCT-3’ |  |  |
| Ala-slit  riboprobe | slit F | 5’-GGCACATAGCTCCGTTAGCAC -3’ | 464 | OM365990 |
|  | slit R | 5’- ACATGCAGAGATTTGAGTAACAACAAG -3’ |  |  |
| Ala-robo1  riboprobe | robo1 F | 5’- CTCTCACGGAACTGGGTCAC-3’ | 795 | OM365991 |
|  | robo1 R | 5’- CCCACAGAATCGTGCCCATA-3’ |  |  |
| Ala-robo2  riboprobe | robo2 F | 5’-GGCACATAGCTCCGTTAGCAC -3’ | 703 | OM365992 |
|  | robo2 R | 5’-GGCACATAGCTCCGTTAGCAC -3’ |  |  |
| Hau-slit  sqRT PCR | slit sqF | 5’- CAATCTCCCAGTCGGTGTCT-3’ | 246 |  |
|  | slit sqR | 5’- AGGAACAGCCACTTCATGCT-3’ |  |  |
| Hau-robo1  sqRT PCR | robo1 sqF | 5’-CGATGAAAATTGACGGTGTG-3’ | 157 |  |
|  | robo1 sqR | 5’-GAACTCGACTGGACGGAGAG-3’ |  |  |
| Hau-robo2  sqRT PCR | robo2 sqF | 5’- CAAAGCTCCTCCGTTACGTC-3’ | 233 |  |
|  | robo2 sqR | 5’- CCGTTGTGATGTTGTTTTGG-3’ |  |  |
| slit-RNAi | S-BamH1F | 5’-GAGGATCCTGCAAACAACACGACTGCAAGAAC-3’ |  |  |
|  | S-Spe1R | 5’-GAACTAGTCGATAGCTGCAAGGTGATATTGTT-3’ |  |  |
|  | ANS-Xho1F | 5’- GACTCGAGCGATAGCTGCAAGGTGATATTGTT-3’ |  |  |
|  | ANS-EcoR1R | 5’- GAGAATTCTGCAAACAACACGACTGCAAGAC-3’ |  |  |
